# A fully integrated whole-head helium OPM MEG: a performance assessment compared to cryogenic MEG

**DOI:** 10.3389/fmedt.2025.1548260

**Published:** 2025-04-04

**Authors:** Maxime Bonnet, Denis Schwartz, Tjerk Gutteling, Sebastien Daligault, Etienne Labyt

**Affiliations:** ^1^ Lyon Neuroscience Research Center, INSERM UMRS 1028, CNRS UMR5292, Université Claude Bernard Lyon 1, Université de Lyon, Lyon, France; ^2^ MEG Department, CERMEP-Imagerie du Vivant, Lyon, France; ^3^ MAG4Health, Grenoble, France

**Keywords:** simulation, OPM, MEG, SQUID, helium OPM, neuroimaging

## Abstract

Magnetoencephalography (MEG) is a neuroimaging technique that measures neuronal activity at a millisecond scale. A few years ago, a new generation of MEG sensors emerged: optically pumped magnetometers (OPMs). The most common OPMs use alkali atoms as the sensing element. These alkali OPM sensors must be heated to approximately 150°C, in contrast to classical MEG sensors [superconducting quantum interference device MEG], which need to be cooled down to −269°C. This article focuses on a new kind of OPM that uses Helium-4 gas as the sensing element, which solves some disadvantages of alkali OPMs. ^4^He-OPM sensors operate at room temperature, with negligible heat dissipation (10 mW) and thus do not need thermal insulation. They also offer a large dynamic range (±200 nT) and frequency bandwidth (2,000 Hz). The main goal of this study is to characterize the performance of a whole-head MEG system based on ^4^He OPM sensors (^4^He OPM MEG). We first simulated different sensor configurations with three different numbers of channels and three different head sizes, from child to adult, in order to assess the signal-to-noise ratio and the source reconstruction accuracy. Experimental testing was also performed using a phantom to simulate brain magnetic activity. The simulation and experiments show equivalent detection capability and localization accuracy on both MEG systems. These results illustrate the benefit of ^4^He OPM sensors that operate at room temperature and are positioned closer to the scalp.

## 1 Introduction

Magnetoencephalography (MEG) (1) is a powerful, non-invasive imaging technique in neuroscience. MEG offers a very good time resolution with a good spatial resolution, particularly when compared to electroencephalography (EEG) (2). MEG records the faint magnetic fields generated by large populations of neurons in the brain. These magnetic fields are of the order of a few hundreds of femtotesla and thus require ultrasensitive sensors. Until now, superconducting quantum interference devices (SQUIDs) were the only sensors available to map the brain’s magnetic field with adequate sensitivity. However, these sensors introduce several practical limitations that are mainly linked to the very low temperature required to achieve superconductivity. Most SQUID sensors used in classical MEG work in liquid helium at −269°C (3). Thermal insulation, i.e., housing the sensors in a cryogenic dewar, makes the SQUID-MEG system cumbersome, and the helmet and sensor array is rigid. Therefore, the distance between the sensors and the scalp is about 2 cm for adults and increases significantly for children and toddlers given their smaller head size as compared to the fixed size of the SQUID helmet. Finally, the need to keep the sensors at a very low temperature requires large energy consumption, which, combined with helium evaporation and the complexity of cryogenic technology, makes the system very costly to run.

Currently, a new kind of MEG sensor has emerged: optically pumped magnetometers (OPMs) (4, 5). In the MEG field, commonly available OPMs use a gas of alkali atoms, usually rubidium, as the sensitive element. A cell filled with the gas is traversed by a laser. The modulation of the intensity of the laser going through the cell is related to the local magnetic field. With such sensors, the magnetic field can be measured in three different orientations, with a noise floor from 15 fT/√Hz to 23 fT√Hz on all axes (https://quspin.com/products-qzfm/) (6). To achieve the Spin Exchange Relaxation Free (SERF) operating mode of alkali OPMs, the cell filled with alkali gas needs to be heated to 150°C. Thermal insulation and air flow are required, so sensors are placed a few millimeters from the scalp. Simulations show a potential 5-fold increase in sensitivity and better source reconstruction and spatial resolution with an alkali OPM compared to a SQUID-based MEG system (7). Numerous studies have assessed the performance of alkali OPMs (7, 8) on healthy volunteers (9–13) and patients (14–16). The results demonstrated the excellent capabilities of these new sensors to record physiological and pathological brain activity. OPM recordings also have several key advantages over classical MEG. For example, the spatial sampling of the signal is greater, potentially allowing a better spatial resolution (17). With OPM sensors being closer to the scalp, the relative position between the sensors and the brain is more preserved if the subject's head moves inside the helmet, although this remains an issue as alkali OPM helmets are also rigid. SQUID MEG systems are far less tolerant to referential changes related to head movements inside the helmet, which can modify the activity topography and amplitude. In addition, the versatility of OPMs means they have a wider range of applications compared to SQUID sensors, which impose stronger technical constraints. For instance, spine or retina recordings have been reported with OPMs (18, 19). However, these alkali OPM sensors are still limited by the need to be heated to be operated and by a limited frequency bandwidth and dynamic range of DC-120Hz and 15 nT at best, respectively (6) (https://quspin.com/products-qzfm/).

An alternative OPM technology has been developed by MAG4Health (https://www.mag4health.com), using Helium 4 gas as the sensitive element. These OPMs do not need to be heated. Consequently, the need for thermal insulation and the heat dissipation issue requiring air flow disappear, and, thus, the sensor can be placed closer to the scalp than alkali OPMs (20). A flexible helmet is proposed that fits all head shapes and fully preserves the relative position between the sensors and the underlying brain regions during head movements. Moreover, ^4^He OPMs overcome the alkali OPM limitations with a high dynamic range (±200 nT) and a very large bandwidth (DC to 2 kHz). The large dynamic range makes recordings possible without any additional field nulling system and, therefore, the MAG4Health OPM MEG system can be used in an existing or standard magnetic-shielded room (MSR). Thanks to this large dynamic range, even though there are large variations in the environmental magnetic field or variations due to the subject's movements, there will be no saturation of the signal. This broadens the conditions of use of OPM sensors, even though, of course, it is still recommended to conduct the recordings in good conditions to limit the noise associated with the subject’s movements in the recorded signal. The large bandwidth also opens new possibilities to record high-frequency brain activities, for example, the high gamma, ripples, and fast ripples seen in pathological and physiological conditions. The first studies conducted with these new ^4^He OPM sensors provided very encouraging results. The results obtained using simulations to assess signal power and spatial resolution (21) were supported by real recordings conducted with five sensors assessing the signal-to-noise ratio (SNR) of evoked brain activities with two experiments, using a somatosensory and visual stimulation paradigm (22). These first results suggest a good equivalence between ^4^He-OPM MEG and SQUID MEG. Two studies were also performed in epileptic patients, showing the good sensitivity of these sensors when compared to SQUID MEG and invasive EEG brain recording (23) and equivalent sensitivity in “real life” recordings when compared to alkali OPMs, despite a higher intrinsic noise floor (24).

A whole-head system with up to 97 positions has recently been developed with tri-axial ^4^He OPM sensors. The sensitivity of ^4^He-OPMs improved, with initial results showing a sensitivity around 50 fT√Hz. Recent results show that ^4^He OPM sensors can now reach a sensitivity better than 30 fT/√Hz on two of the three axes, with a very limited 1/f noise rise only visible below 5 Hz on raw data.

In this study, our main goal was to assess the performance of this whole-head system compared to SQUID MEG. As in several studies on alkali OPM sensors (7, 8) and ^4^He OPM sensors (21), we used simulations as a first step of performance assessment. The simulations were based on realistic “real life” noise floor and sensor positions. After carrying out detailed noise floor measurements across several days, we carried out simulations evaluating the SNR and source reconstruction accuracy with various sensor array configurations and head sizes. This step allowed us to estimate the statistical errors. In the second step, we evaluated systematic errors on a MEG system by using a phantom containing current dipoles that allowed us to generate artificial MEG signals with minimum noise.

## 2 Methods

### 2.1 MEG systems overview

#### 2.1.1 ^4^He-OPM MEG whole head system

The whole-head ^4^He-OPM system, built by MAG4Health, has 96 tri-axial sensors (288 channels) covering all of the head. The headcap is flexible, so it can fit all head shapes and sizes. This headcap exists in an adult size with up to 97 positions and in a child size with up to 89 positions. A tightening system makes it possible to adjust the sensor array on the subject's head. Each sensor measures the brain's magnetic field along one radial axis (R) and two tangential axes (T). The barycenter of the magnetic field measurement in the Helium gas cell is located 3.2 mm from the scalp surface, as evaluated from the decay of the magnetic field of a dipole (25).

The ^4^He-OPM sensors rely on the parametric resonance of ^4^He atoms in a near-zero magnetic field (26). The brain magnetic field measurement consists of the measurement of light intensity modulation caused by the dynamics of the electronic spin of the ^4^He atoms.

For this measurement, ^4^He atoms need to be brought to their first excited state 2^3^S_1_, called metastable, using a high-frequency plasma discharge. This discharge dissipates 10 mW for a 0.8 cm^3^ cell, compared with ∼700 mW dissipated by the heating required for a 0.008 cm^3^ cell in alkali OPM. Then, this metastable level is optically pumped using the D_0_ (2^3^S_1_ → 2^3^P_0_) transition with a laser. Unlike most OPMs, where the pumping light is circularly polarized, ^4^He-OPM sensors use linearly polarized light. This results in a spin polarization of ^4^He atoms called alignment (25).

The cell filled with the helium gas measures 1 cm in diameter and 1 cm in height. This cell is surrounded by three orthogonal Helmholtz coils to apply the radio-frequency fields also used in the real-time self-compensation of the magnetic field offset along three axes of the sensor (closed loop operating mode).

This ^4^He-OPM system achieves a dynamic range of ±200 nT with a sensitivity better than 30 fT/√Hz on two out of three axes (one radial and one tangential) and 200 fT/√Hz for the third axis, allowing recording in an MSR without any field nulling system. The bandwidth ranges from DC to 2 kHz (27), enabling the recording of the full range of brain oscillatory activity up to ripples and fast ripples.

#### 2.1.2 SQUID MEG

The classical MEG system (SQUID MEG) used in our comparison was a CTF MEG system (CTF MEG Neuro Innovations INC., Port Coquitlam, Canada), with 275 axial gradiometers located between 1.8 and 3 cm from the scalp of an adult head. This system has a noise level of approximately 5 fT/√Hz, which is the typical noise for a SQUID MEG system.

### 2.2 Empty room recordings

To run the simulations with a realistic sensitivity for the new ^4^He OPM MEG, a determination of the typical experimental noise level of the system was necessary. Thus, several empty room recordings were conducted in the MSR of our CTF MEG system (two *μ*-metal layers and one copper layer, Vacuumschmelze, Hanau, Germany). The ^4^He OPM MEG was installed on the chair of the classical MEG system at the center of the MSR. The signal was recorded with a 47-sensor configuration with a sampling rate of 1,003.2 Hz for 1 min, at different times of the day (12:30 a.m., 3:30 p.m., and 4:00 p.m.). In addition, a long recording of 20 min with a sampling rate of 3 kHz was also performed (5:30 p.m.) to check the signal stability. Power spectrum densities were then computed without any denoising processing of the data using Welch's method in the Python NumPy toolbox (28).

### 2.3 Performance evaluation

#### 2.3.1 Statistical and systematic errors

In this study, to characterize the sensors’ performances, we evaluated the statistical errors and systematic errors.

Statistical errors arise from random, unknown, and uncontrollable events: in our case, all sources of magnetic fields not linked to brain activity, from intrinsic sensor noise to ambient noise in the MSR. This error can be evaluated thanks to simulations using random realizations of the noise shaped to reproduce the actual noise spectrum measured on the system (see section 2.3.2).

Systematic errors are reproducible errors corresponding, among others, to imperfect manufacturing of the phantom used for tests, imperfect models of the sensors, or hypothesizing an inaccurate measurement barycenter. These errors can be evaluated by making all sources of statistical errors negligibly small, for instance, by generating a large enough MEG signal in a phantom (see Section 2.4). This allows reaching a very high SNR in order to characterize only these systematic errors.

#### 2.3.2 Statistical errors: simulations modalities

##### 2.3.2.1 Head model

To cover a range of head sizes from infant- to adult-sized heads, simulations were conducted on three different head sizes: the standard head with a circumference of 58 cm (H58); a middle size of 55 cm (H55), which represents a child's head at 6 years old; and a small size of 50 cm (H50), which represents an infant head size.

T1-magnetic resonance imaging (MRI) obtained from a volunteer was used to create these three realistic head models by scaling down the standard head (H58). FreeSurfer software was used to segment the brain, skull, and scalp surfaces (29). A mesh of the gray-white matter interface was created with nearly 130,000 vertices on each hemisphere.

##### 2.3.2.2 ^4^He-OPM MEG sensor layouts

To test for the effect of the number of sensors, the simulations were performed using three different sensor layouts, i.e., 97 sensors (maximum number of slots on the current headcap), 63 sensors, and 48 sensors, with an even distribution of the sensors on the head in each. Each layout was simulated on the adult and child head sizes. For the smaller head size (H50), the simulations used three other sensor layouts: 48 sensors [which is the maximum number of sensors that can be placed on a small head (H50) due to the ^4^He sensor's size], 24 sensors, and 16 sensors. The goal was to compare, for a given head size, the performance achieved with the different sensor arrays with that obtained with the standard SQUID array.

##### 2.3.2.3 Source space

To define the source space used in the simulations, the original cortex surface mesh was subsampled to 4,098 vertices per hemisphere. Considering this number of vertices, the mesh resolution was sufficient to be well fitted to the cortical folding. Each vertex was used to set a dipole source position and orientation (normal to the local surface of the cortex). This resulted in a source space of 8,196 dipoles. Consequently, among the 8,196 dipoles constituting the distributed source space, most of the dipoles were either tilted or tangential to the head surface. The resulting field modeled from this source space led to an external field that simulated realistic MEG signals.

The analysis was focused on cortical sources located at a maximum distance of 20 mm from the scalp for the H58 head size. This source subset includes various dipole orientations and all these sources are in the ideal sensitivity volume for both SQUID MEG and ^4^He-OPM MEG (30). For the other head sizes (H55 and H50), the source spaces used the same source indexes as the ones selected for the H58. Therefore, the number of cortical sources was strictly the same for all the head sizes.

##### 2.3.2.4 Head-sensor alignment

###### 2.3.2.4.1 ^4^He OPM MEG

We used real 3D positions of the ^4^He OPM MEG whole-head system on the H58 head to define the location of each ^4^He OPM sensor for the simulations. To register these positions, the following procedure was used:

1. A 3D Polhemus digitizer (Colchester, USA) was used to locate each ^4^He OPM MEG (97) with respect to the location of three fiducial points (nasion, left tragus point, and right tragus point). Three points were digitalized for each sensor position, allowing us to compute the exact location on the scalp and calculate the exact orientation of the radial and two tangential axes.
2. The sensors were projected onto the scalp at the nearest location.
3. All the sensors were then shifted to 3.2 mm above the scalp in the radial direction to fit accurately the geometry and position of the sensing cell.

**Figure 1:**
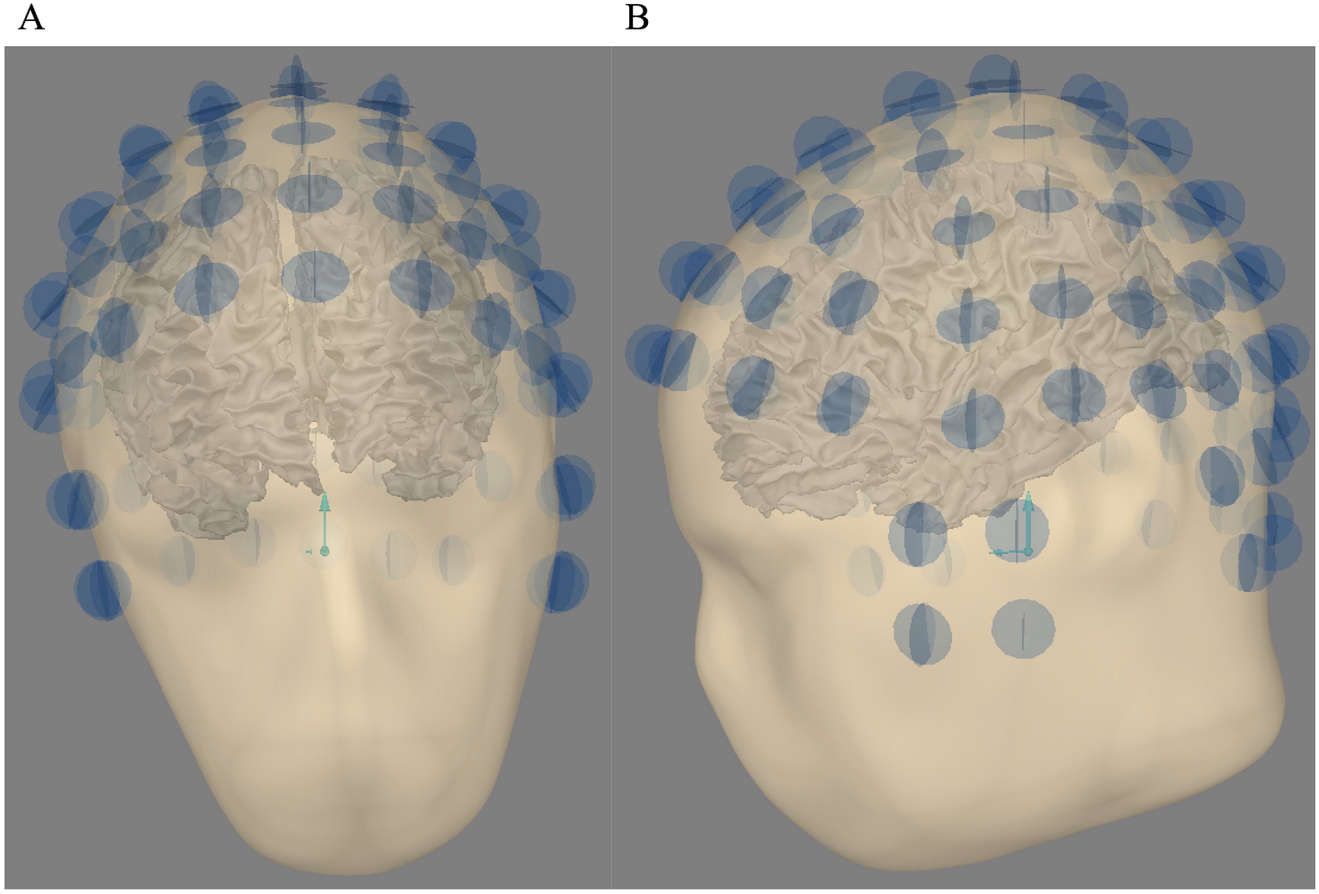
**(A)** Head sensor alignment for ^4^He OPM MEG—coronal view. **(B)** Head sensor alignment for ^4^He OPM MEG—sagittal view. For each OPM sensor (97), the two orthogonal circles show the two recording axes used in this study (one radial, one tangential).

Figure 1 shows the alignment between the head and the sensors. For each OPM sensor, two blue orthogonal circles showed the two recording axes used in this study, one tangential and one radial. For the three head sizes, the relative sensors' positions remain equivalent.

###### 2.3.2.4.2 The SQUID MEG system

**Figure 2:**
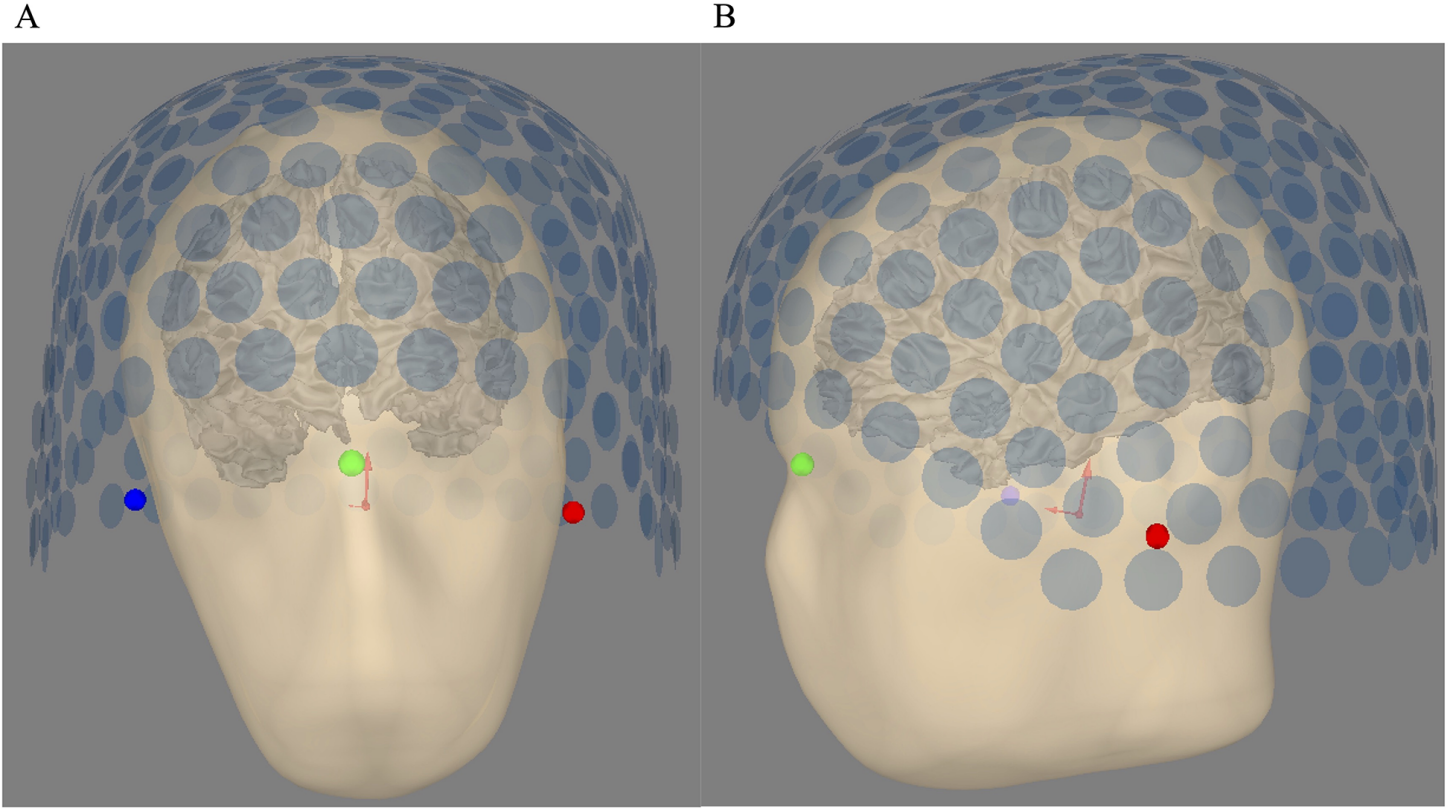
**(A)** Head sensor alignment for SQUID MEG—coronal view. **(B)** Head-sensor alignment for SQUID MEG—sagittal view. The bullet points are the three fiducial points, i.e., the left tragus, the nasion, and the right tragus, which are red, green, and blue, respectively. Each blue circle shows the location of the first pickup coil of the 275 CTF gradiometers.

For the SQUID MEG system, the T1-MRI of the standard head (H58) was coregistered using the MRI fiducials and the real localization of the SQUID MEG sensors obtained thanks to the head position indicator (HPI) coils localized on the three fiducial points. The positions for H55 and H50 were the same as for H58. Figure 2 shows this coregistration between the SQUID MEG sensors configuration with the subject’s head.

##### 2.3.2.5 Simulation

The sensitivity of the ^4^He OPM is optimal on two of the three axes, so all the analyses in this study were performed only on these: one radial and one tangential axis. In all the simulations and analyses presented in this study, the ^4^He OPM MEG was modeled as a magnetometer with one point of integration at the barycenter of the sensitive element.

All analyses were conducted using MNE-Python (version 1.3.1) (31) on Python (version 3.11.2).

To simulate a brain signal, a Gaussian shaped time course (Tc) with a maximum amplitude of 100 nA.m was used to mimic the activation of each source on 600-time samples. The forward model for each source was computed using a boundary element model (BEM) with one layer representing the inner skull and a relative conductivity of 0.3. The MEG signal was computed using the following equation:

(1) M=Tc*Fop+noise

with M denoting the simulated MEG signal (number of sensors×number of time samples) for a given source; Tc denoting the time course of activation; Fop denoting the forward operator for a given source; and noise denoting random noise with a Gaussian distribution, with 35 fT/√Hz spectral density for ^4^He OPM MEG (see Section 3.1) and 5 fT/√Hz for SQUID MEG in accordance with the noise floor of each sensor.

##### 2.3.2.6 Signal-to-noise ratio computation

The SNR was used to evaluate the detection ability of each kind of sensor array. For ^4^He OPM arrays, SNRs were computed on the radial axis or the tangential axis individually or both combined. For a single axis, we use Equation 2 to calculate the $\mathrm{SN}R_{i,j}$ for each source *j* and sensor *i*:

(2) $$\mathrm{SN}R_{i,j}=\frac{\max(S_{i,j})}{BL_{i}}$$

with *S_i,_*_j_ denoting the signal generated by source *j* along time for sensor *i* and BL_i_ denoting the noise level for sensor *i*.

To compute the SNR combining the radial and tangential axis, we used the following equation:

(3) $${\mathrm{SNR}}_{i,j}^{r,t}=\frac{\max(S_{i,j}^{r})+\max(S_{i,j}^{t})}{\left(\sqrt{\sqrt{\left({BL}_{i}^{r^{2}}+{BL}_{i}^{t^{2}}\right)}}\right)}$$

with $S_{i,j}^{r}$ denoting the signal generated by source *j* along time for sensor *i* on the radial axis, $S_{i,j}^{t}$ denoting the signal generated by source *j* along time for sensor *i* on the tangential axis, $BL_{i}^{r}$ denoting the noise level for sensor *i* on the radial axis, and $BL_{i}^{t}$ denoting the noise level for sensor *i* on the tangential axis.

For source *j*, the maximum ${\mathrm{SNR}}_{j}^{r,t}$, is calculated by

(4) $${\mathrm{SNR}}_{j}^{r,t}={\mathrm{MAX}}_{i=1}^{i=nbsensors}{\mathrm{SNR}}_{i,j}^{r,t}$$

with nb_sensors denoting the number of sensors for a given array configuration (OPM: 97, 63, 48, 24, or 16; SQUID: 275).

##### 2.3.2.7 Localization errors

The dipoles’ localization errors were assessed using simulated signals from one tangential axis and the radial axis together. Consequently, the computed forward operator included information for both axes. The MNE-Python function “fit_dipole” was used to calculate the dipole localization using the simulated brain signal at the peak amplitude of the source activation. The noise covariance was computed from the random noise described in Section 2.4. The process was repeated 100 times for each source by computing a new MEG signal (M) from Equation 1 with a new noise draw. The accuracy of the dipole localization was evaluated through the dipole localization error (DLE), which is the Euclidean distance between the actual source position and the estimated dipole position.

##### 2.3.2.8 Statistical comparisons

SNR and DLE values were compared between SQUID and OPM with a repeated measures ANOVA with the following factors: sensor type (OPM48/OPM64/OPM97/SQUID for H58 and H55 head sizes; OPM16/OPM24/OPM48/SQUID for H50 head size) and measures (SNR/DLE): F(4, 1,556) all *p* < 0.01. *Post hoc* analyses were then performed for comparisons between each OPM array configuration and SQUID.

#### 2.3.3 Systematic errors: phantom description

The phantom test is a practical way to obtain a reliable estimate of the effects of reproducible imperfections in the measurement and the subsequent postprocessing steps. Two kinds of phantoms exist: dry phantom or fluid phantom. The fluid phantoms are typically glass containers filled with saline solution. Dipolar sources are inserted within the container and energized through a coaxial cable or a twisted insulated wire. As outlined by Ilmoniemi (32), a major issue with the fluid phantom is that the insulating structures near the dipole may distort the volume current, making the phantom useless as an absolute calibrator. Therefore, we chose to test our measurement system with a dry phantom. We also chose a spherical geometry for our phantom as the improved precision in estimating cerebral sources obtained with realistically shaped conductor models and a fluid phantom is small (33). Moreover, spherical geometry also provides an easy way for reproducing the current source by using isosceles triangle coils with their vertex at the center of the sphere (32). These coils produce the same magnetic field as the original tangential primary current and its associated 3D distributed volume current. In this study, as the SQUID MEG machine only recorded the radial component of the magnetic field, we have chosen to only consider the radial axes of the ^4^He OPM sensors for comparison.

**Figure 3:**
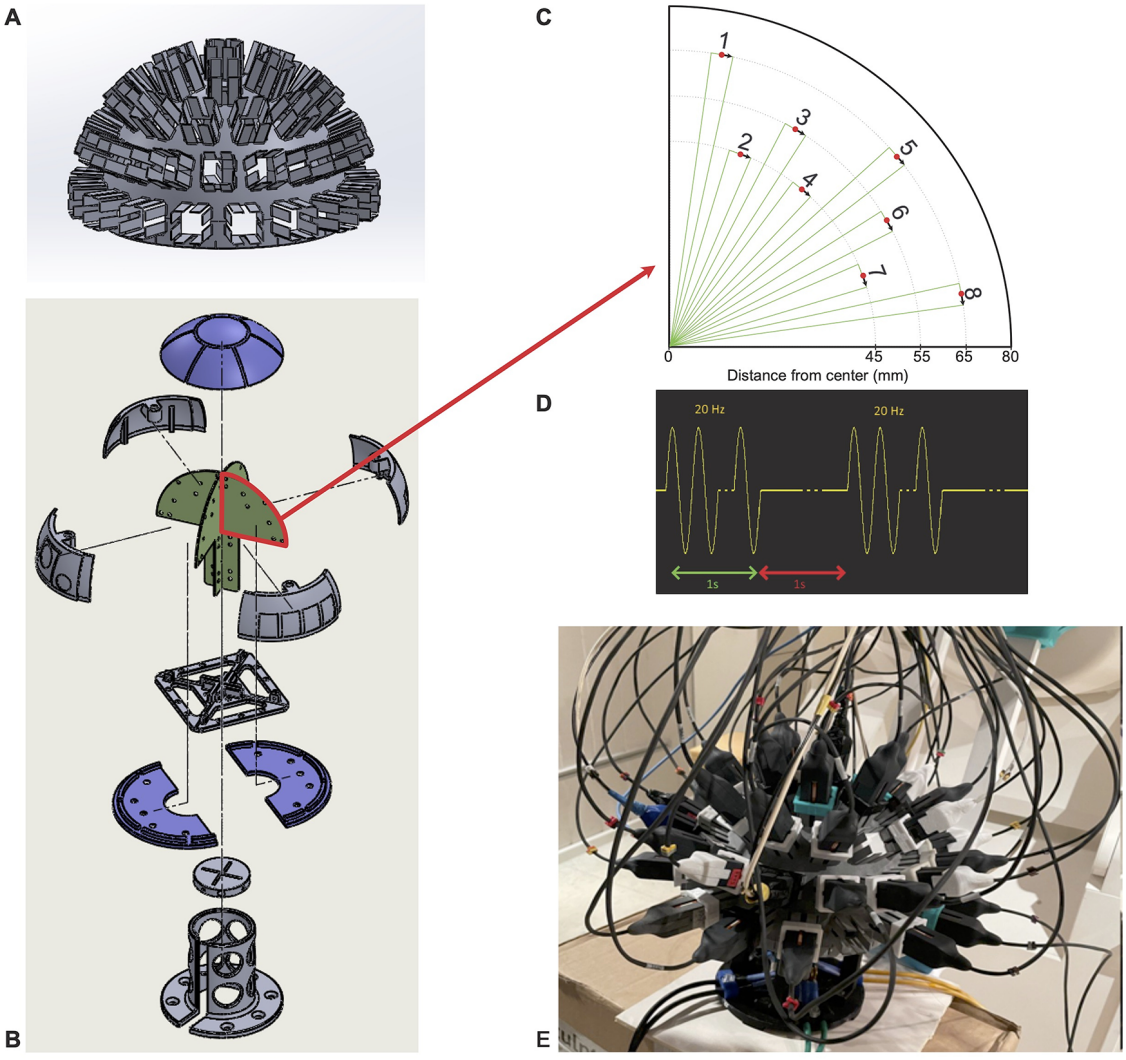
Phantom description. **(A)** Helmet where the OPM sensors were positioned in 45 slots. **(B)** Phantom schematic with the four printed circuit boards (PCBs) where the dipoles were located in green. **(C)** Schematic depiction of the real position and orientation of the dipoles numbered from 1 to 8 on the PCBs. **(D)** Excitation signal used for each dipole (1 s of stimulation at 20 Hz and 1s of rest repeated 20 times). **(E)** Picture of the real phantom with the helmet on and the sensors filling the 45 available slots.

The phantom used in this study consists of a one-half sphere with a radius of 8 cm hosting four printed circuit boards (PCBs). Each PCB has eight isosceles triangle printed tracks as current dipoles at different locations (see Figures 3B,C), and the dipole lengths (bases of the triangles) are 5 mm in length. A “hemispherical helmet” with 51 evenly spaced slots was fitted on this phantom and allowed placing the ^4^He OPM sensors as shown in Figures 3A,E. For the phantom recordings, 47 sensors were used.

For the SQUID MEG recording, the phantom was fitted in the SQUID MEG helmet. The HPI coils allowing the localization of the phantom within the SQUID MEG were fitted on the hemisphere at the “nasion” (*z* = 0, azimuth 0°), “left ear” (*z* = 0, azimuth 90°), and “right ear” (*z* = 0, azimuth −90°) positions. The CTF SQUID MEG sensors are not always set in positions where they measure only the magnetic field radial to the sphere, and this is particularly true for the lowest sensors of the helmet. Thus, in this study, only the four dipoles at the very top of the phantom were used in order to have a fair comparison between the OPM and SQUID systems.

To reduce the statistical error, a high dipole value of 2,000 nA.m and a high number of repetitions were used. Each current dipole, one by one, was activated with a sinusoidal current at 20 Hz for 1 s followed by a null current for 1 s. This sequence was repeated 20 times (as shown in Figure 3D). Thus, we had 400 repetitions of sinusoids for each dipole.

The systematic errors were evaluated as follows. To estimate each dipole location, the MNE-Python function “fit_dipole” was used on the average of the 400 sinusoids at the latency of the maximum amplitude. The noise covariance for each MEG system was computed from two empty room recordings, one for the ^4^He OPM MEG system and one for the SQUID MEG system, respectively. The DLE was estimated by computing the distance between the estimated and the actual position for each dipole.

## 3 Results

In this study, after estimating the real noise floor of the ^4^He OPM system, the signal-to-noise ratio and source localization accuracy were assessed through simulations and compared to those obtained with classical SQUID MEG. This provides an estimate of the statistical errors. In addition, a phantom setup was used to evaluate systematic localization errors of the whole-head ^4^He OPM MEG device and these were compared to the SQUID MEG results.

### 3.1 OPMs empty room recordings

**Figure 4:**
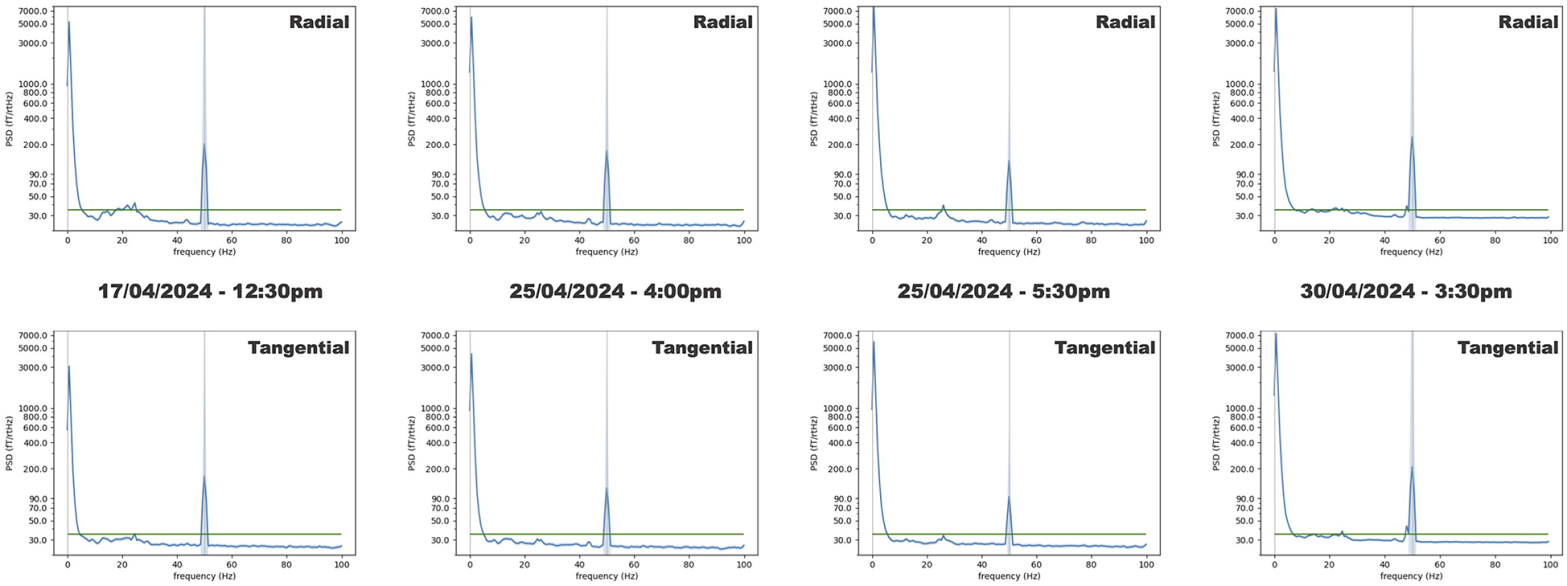
Empty room PSDs averaged over all sensors. The top row shows the PSDs computed for the radial component of the magnetic field. The bottom row shows the PSDs computed for one of the tangential components of the magnetic field. The green lines show the 35 fT/√Hz level. The shaded areas show the standard deviation. From left to right, the three first recordings (1 min long) were performed at 12:30 p.m., 4:00 p.m., and 5:30 p.m., with a week of delay between the first recording and the next two. The last recording (17 min long) was performed 5 days later at 3:30 p.m.

Figure 4 shows the averaged power spectral densities (PSDs) computed from the empty room recordings. The averaged PSDs mostly show values under 35 fT/√Hz, with average values between 6 and 44 Hz: radial 30.06 fT/√Hz ± 1.53 and tangential 29.32 fT/√Hz ± 1.48. Amplitude variations were noted around 20 Hz, which is in line with the natural resonance frequency of the building hosting the MEG lab. Notes taken during the recordings showed that increased power around 20 Hz was closely linked to car and helicopter movements around the MEG lab. Given these results, we chose to use 35 fT/√Hz as the noise floor for the ^4^He OPM MEG system in our simulation as the worst-case scenario for the ^4^He OPM MEG, avoiding an overestimation of its performance.

### 3.2 Statistical errors

#### 3.2.1 SNR results

**Figure 5:**
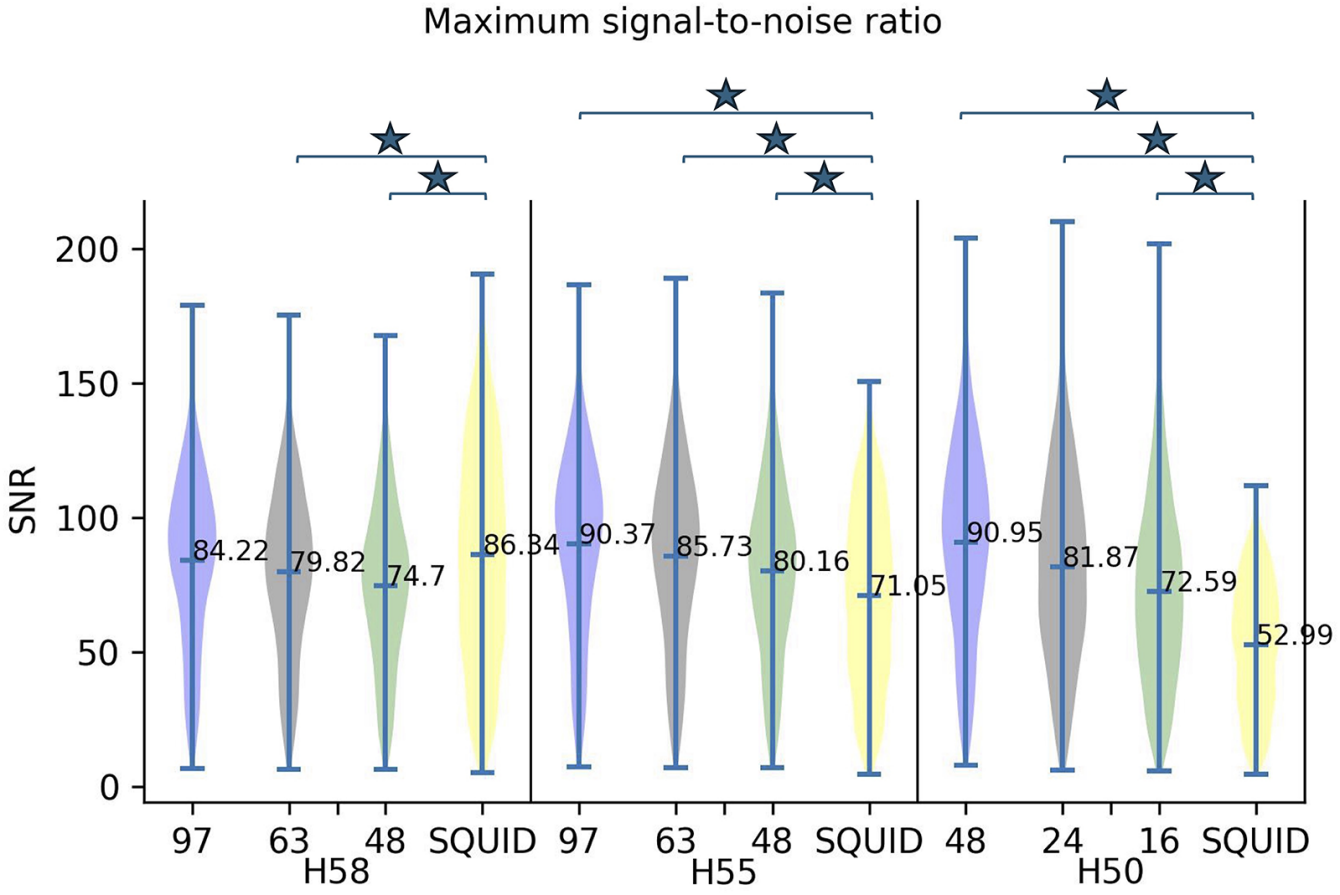
Maximum SNR distribution for the three sensor configurations for 4He OPM MEG, i.e., 97 (blue), 63 (gray), and 48 (green) sensors, and SQUID MEG (yellow) on head sizes H58 and H55. For H50, the three sensor configurations were 48 (blue), 24 (gray), and 16 (green) and the SQUID (yellow) configuration. For the OPM configurations, the max SNR was calculated with a combination of the two axes (tangential + radial). The mean of each distribution is shown at the horizontal marker. Horizontal bars and asterisks highlight the statistically significant differences (*p* < 0.01).

Figure 5 shows the maximum SNR (Equation 4) distribution combining the tangential and radial axes together (Equation 3) on the three sensor array configurations with the ^4^He OPM MEG on the H58 and H55. For the H50, the 48-, 24-, and 16-sensor array configurations are displayed. The maximum SNR obtained with SQUID MEG for the three head sizes is given for comparison.

^4^He OPM MEG shows a slightly lower maximum SNR compared to SQUID MEG for the H58 head. For this head size, the maximum SNR of the 97-sensor configuration was not significantly different from the SQUID MEG SNR. As the head size decreases, the SNR for the OPMs increases and becomes significantly (*p* < 0.01) higher than the SQUID MEG SNR (for all OPM sensor arrays) because the OPM sensors are closer to the brain. The head size effect is very clear, especially for H50, where the SNR of the ^4^He OPM MEG with 48 sensors is 60% higher than the SQUID MEG SNR (90.95 compared to 52.99).

The maximum SNRs computed for the radial or tangential axis only are provided in the Supplementary Materials (Supplementary Figure 1). The maximum SNR for both combined axes is improved as compared to that computed for the radial or tangential axis only.

#### 3.2.2 Dipole localization errors

**Figure 6:**
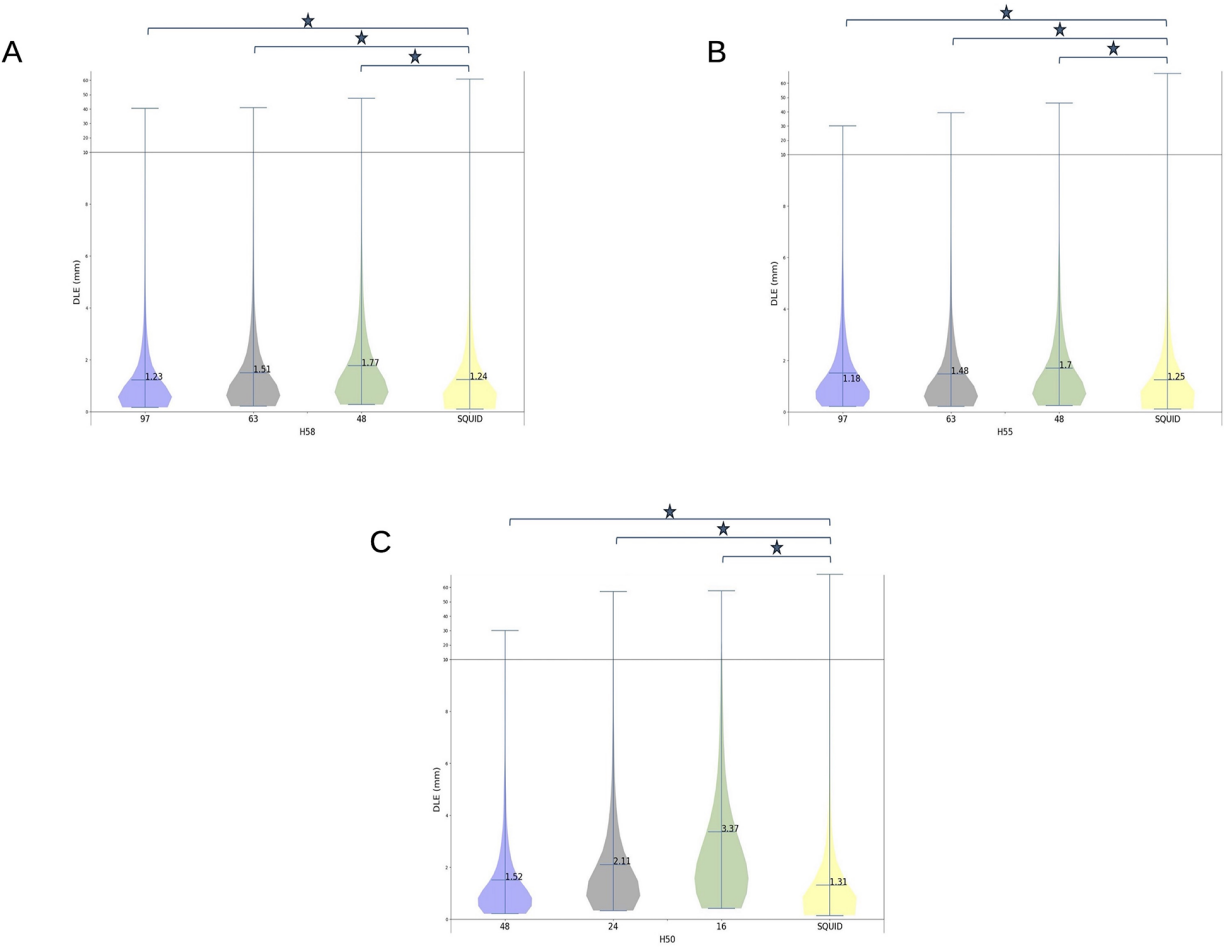
Dipole localization errors (DLEs) for the three sensor configurations for 4He OPM MEG, i.e., 97 (blue), 63 (gray), and 48 (green) sensors, and SQUID MEG (yellow) on head sizes H58 **(A)** and H55 **(B)**. For H50 **(C)**, the three sensor configurations were 48 (blue), 24 (gray), and 16 (green) and the SQUID (yellow) configuration. The mean of each distribution is shown at the horizontal marker. The horizontal line in the plot shows a change of scale for the vertical axis. Horizontal bars with an asterisk indicate statistically significant differences (*p* < 0.01).

Figure 6 shows the DLEs for the four configurations of the sensor array (97, 63, 48 ^4^He OPM and SQUID) for head sizes H58 and H55. For H50, the comparison was based on the 48, 24, and 16 ^4^He OPM sensor array configurations with respect to the SQUID array. This figure illustrates, for a given head size, the comparison of three OPM sensor arrays to a standard SQUID array. However, a comparison of one sensor array between the different head sizes was not valid, as the simulations were performed independently. If the source spaces had the same number of sources, the geometry of the source spaces could not be strictly similar between the different head sizes.

For the H58 (adult) and H55 (child) head sizes, the DLE achieved with the 97 ^4^He OPM sensors array was significantly (*p* < 0.01) lower (better) than the DLE obtained with the SQUID MEG machine. However, the ^4^He OPM MEG DLE for the 48- and 64-sensor arrays were significantly (*p* < 0.01) higher than that of the SQUID. However, for these configurations with a lower number of ^4^He OPM sensors, the DLE was only 2–5 tenths of a mm higher than the SQUID DLE.

For the H50 head size (small children), the DLE was significantly different (*p* < 0.01) between the OPMs and SQUID for all OPM sensor arrays. The 48-sensor configuration achieved a better DLE with a difference of only 0.21 mm as compared to the SQUID machine. However, compared to the 16-OPM-sensor configuration, the SQUID array provided a far better source reconstruction accuracy.

### 3.3 Systematic errors: phantom recordings

**Figure 7:**
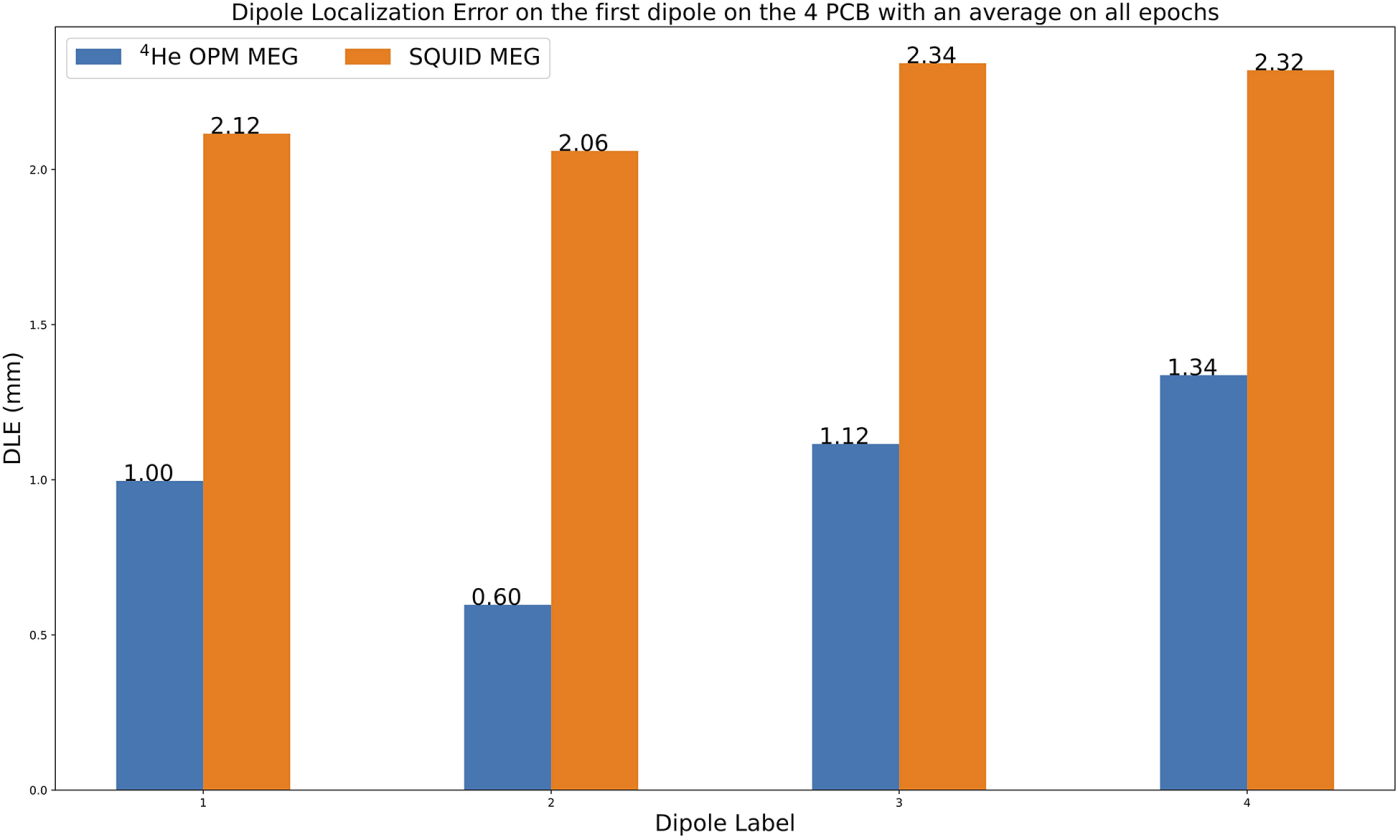
Average dipole localization errors (DLEs) between actual and estimated dipoles (in mm) for the two MEG systems for all epochs. The mean of each distribution is shown at the horizontal marker.

Figure 7 shows the average DLE for the ^4^He OPM MEG and the SQUID MEG of all 400 epochs for the four top dipoles of the phantom. Based on the “fit_dipole” MNE method used in this work, the localization errors ranged from 0.60 to 1.34 mm for the ^4^He OPM MEG, whereas for the SQUID MEG, the DLE ranged from 2.06 to 2.34 mm. The dipole localization errors from all dipoles of the phantom are provided in the Supplementary Materials (Supplementary Figure 2).

## 4 Discussion

The current literature and the results presented here show that the ^4^He OPM MEG sensors can overcome some disadvantages of classical SQUID MEG. The main limitation solved with OPM MEG is the fact that the sensors can be placed on the scalp. This is especially true with ^4^He OPM MEG, for which there is no heat dissipation issue. This provides access to a higher signal amplitude when compared to SQUID MEG. This higher amplitude, combined with the measured sensitivity of ^4^He OPM MEG of 30 fT/√Hz on two of the three axes, allowed the ^4^He OPM MEG system to have an overall equivalent or better performance when compared to a SQUID system for cortical sources. The maximum SNR measured using ^4^He OPM MEG showed equivalent or better results than SQUID MEG for the small head sizes, i.e., H55 and H50. This advantage of OPM MEG was also reported in several papers (7, 34). Regarding dipole localization accuracy, a similar trend was also observed with greater accuracy for the smaller heads with ^4^He OPM compared to SQUID. This was also reported with alkali OPMs (35). The phantom tests show that ^4^He OPM MEG yields a lower systematic error than that obtained with SQUID MEG.

### 4.1 Empty room recordings and the ^4^He OPM MEG noise floor

The results from the empty room recordings demonstrate the improvement in the sensitivity of the current ^4^He OPM. The first proof-of-concept sensors had 210 fT/√Hz (20), the first recordings on healthy volunteers 45 fT/√Hz (22), and 30 fT/√Hz was found in the current study. The results also showed the stability of this figure, which remained reproducible across the empty room recordings conducted at different times during the same day, across weeks, and during long recordings. This reproducibility testifies to the reliability of these sensors, which do not suffer from the alkali reactivity with glass and buffer gas or thermal effects related to repetitive heating and cooling of the cell observed in alkali OPMs (36). Note that these results were obtained in a regular (two mu metal layers and one aluminum layer) MSR without any active magnetic field compensation or post-processing. Thus, the ^4^He OPM sensitivity approaches that of alkali OPMs, which is notable if we consider the kind of MSR and the absence of denoising for the measurement reported in this paper as compared with the values previously reported for alkali OPMs (6, 35).

### 4.2 Statistical errors: SNR simulations

These real-life empty room recordings allowed us to determine a realistic noise floor for our simulations. The simulations were also performed with a realistic setup based on the true geometry of the headcap, which is currently part of the MAG4Health product. The noise level measured in real conditions was 30 fT/√Hz on average on both axes. However, simulations were conducted with the sensitivity set to 35 fT/√Hz so as not to overestimate the performance of the ^4^He OPM MEG system. The range of SNR values obtained through the current simulations was equivalent to those obtained experimentally in a previous study (22).

Overall, the results are in line with the current literature showing that OPMs have a clear advantage over SQUIDs when the head size is smaller but provide equivalent or lower SNRs for adults (7, 21), mainly due to their more limited sensitivity (still 3–4 times lower than SQUID). The number of sensors in the headcap (48, 64, or 97) did not significantly change the results for head sizes H58 and H55, meaning that the spatial sampling of the simulated signal did not affect the SNR measures, even though we focused our analysis only on cortical sources (less than 2 cm from skin surface). Furthermore, combining both axes improved the maximum SNR. This reveals that the radial and tangential axes provide complementary information.

### 4.3 Statistical errors: dipole localization errors in the simulations

Several studies have investigated the theoretical performance of OPMs for source localization with various strategies and error measures (21, 37–41). As shown by the SNR measures, irrespective of the OPM type, the reduced sensitivity of OPMs is counteracted by their closer proximity to the brain. They may achieve equivalent or better localization accuracy for populations of neurons relatively close to the scalp (<2 cm). This advantage is also obvious when the size of the head decreases. In this study, a simple but robust measure of accuracy was used: the dipole localization error. The ^4^He-OPM MEG with 97 sensors showed equivalent or better accuracy than SQUID MEG, with better performance for smaller head sizes. When the number of OPM sensors was lower (63 or 48), the accuracy of OPM MEG was a few tenths of a millimeter worse than SQUID MEG for the 100 nA.m dipoles used in these simulations. This should not compromise the few-millimeter precision that is needed for most MEG applications. It is interesting that, for the smaller head (H50) with 48-sensor configuration, a near equivalent accuracy to SQUID MEG was achieved, a result particularly meaningful for future research in infants. A similar finding was reported in Ref. (7), where the DLE was estimated while adding the percentage of sensors.

### 4.4 Systematic errors: dipole localization errors with the phantom

The results obtained in the phantom test with the four current dipoles show a better localization accuracy with ^4^He-OPM MEG in the 47-sensor configuration compared to SQUID MEG. The systematic error for the SQUID system may be explained by the SQUID sensor array geometry not perfectly fitting the spherical shape, as a perfect fit guarantees the measurement of the sole radial component of the magnetic field. Indeed, the bottom dipoles of the phantom were detected with a DLE above 1 cm. For this reason, we chose to keep only the four top phantom dipoles for the comparison with the ^4^He OPM system, but in spite of this choice, the error remained quite high. This higher systematic error may partially arise from errors in HPI coil placement, which may cause a shift in the alignment between the phantom and the SQUID sensor array.

Compared to the previous results reported in a phantom experiment with alkali OPMs (35), the dipole localization accuracy achieved in this study was better. However, the experimental phantom setups were different. Boto et al. (35) used a spherical phantom with saline solution, one dipole, and a 25 triaxial alkali OPM. With this setup, by using only radial axis data for the dipole fitting, they achieved a dipole localization accuracy of 5.11 mm. A more recent study (42) on a realistically shaped phantom with electrolytic fluid reported a dipole localization error of 5.51 mm. This study used only a 36-channel OPM to record the radial components of the magnetic fields relative to the head. We obtained better accuracy (ranging from 0.6 to 1.34 mm), but we recorded the dipolar activity with a 47 Helium OPM, and we used a dry phantom. We have reproduced the result on four dipoles in different positions. As the goal of this study was to compare the dipole localization accuracy achieved with the ^4^He OPM array to that obtained with the CTF SQUID MEG machine, we only focused the analysis on the radial axis measurements from the ^4^He OPM array. This could be seen as a limitation. However, in the experimentation with 25 triaxial alkali OPMs, according to the authors, the phantom they used made it possible to record the magnetic field along the three axes of alkali OPMs. However, even when using three axes of information, they did not show any noticeable improvement in the dipole localization accuracy. As discussed by the authors, 's to be expected since, for a dipole in a spherical conductor, the addition of tangential measures would not be expected to offer any extra information. Rather, the triaxial measurement is advantageous due to its ability to reduce interference, boost signal, and ensure uniform coverage. Regarding the study that used a realistically shaped fluid-phantom, unfortunately, the 36-channel OPM array only recorded the radial component of the magnetic field relative to the head. Therefore, the added value of tangential biomagnetic measurement was not possible, even though their phantom offered this possibility. In future research, it would be interesting to investigate the added value of the tangential measurements on the accuracy of dipole localization. This should be done from recordings on a dry phantom and a realistically shaped fluid phantom.

### 4.5 Study limitations

This study has several limitations. The first was the limited subset of sources included in the simulations that explored only the best sensitive volume of MEG. The results presented here cannot be translated to deeper sources, especially in more central areas of the brain where OPM MEG would be disadvantaged, considering its lower sensitivity (5). However, this lower sensitivity could be compensated for by the higher spatial resolution of OPM sensors due to their higher proximity to the scalp surface, allowing better separation of field patterns arising from spatially separated current sources in the brain, such as deep and cortical sources.

The number of sensors seems to be the critical parameter for source reconstruction. An increased number of sensors can help for spatial frequencies, which enable a better spatial resolution and can reduce the leakage effect. However, as a number of studies have shown, increasing the number of sensors may not be the best way to increase the performance of OPM systems, as they reach the same spatial resolutions with far lower numbers of sensors than their SQUID counterparts. Taking the vectorial nature of two- or three-axis measurement for denoising and source localization into account will be a key point for the optimal use of these new systems (11, 43, 44).

Finally, the ^4^He OPM MEG system had not yet incorporated a localization of the sensors at the time the phantom recording was performed. The theoretical position of the sensors on the spherical surface was used in the localization process. The systematic errors that may result from sensor localization errors are thus disregarded in the current data for ^4^He OPM MEG.

## 5 Conclusion

In conclusion, ^4^He-OPM sensors yield an SNR and dipole localization accuracy substantially equivalent to SQUID MEG in most configurations. The results show particularly good performance on smaller head sizes. This underscores the potential of OPM MEG for research on children and toddlers, negating the disadvantages of SQUID MEG, such as a rigid helmet and movement restrictions, by using a flexible headcap that allows close contact with the scalp.

## Data Availability

The raw data supporting the conclusions of this article will be made available by the authors, without undue reservation.
