# Supplementary material for: A fully integrated whole-head helium OPM MEG: a performance assessment compared to cryogenic MEG
Source: Front Med Technol. 2025 Apr 4;7:1548260. doi: 10.3389/fmedt.2025.1548260 (PMC12006120; doi:10.3389/fmedt.2025.1548260)
Supplement: Supplementary file 1 [file Datasheet1.pdf]

Supplementary Material

A fully integrated whole head Helium OPM MEG: performance assessment compared to cryogenic MEG

Maxime Bonnet[1,2], Denis Schwartz[1,2], Tjerk Gutteling[1,2], Sebastien Daligault[1], Etienne Labyt[3*]

[1]Lyon Neuroscience Research Center, INSERM UMRS 1028, CNRS UMR5292, Université Claude Bernard Lyon 1, Université de Lyon, Lyon, France.

[2]CERMEP-Imagerie du Vivant, MEG Departement, Lyon, France

[3]France MAG4Health, Grenoble, France

Supplementary Figures

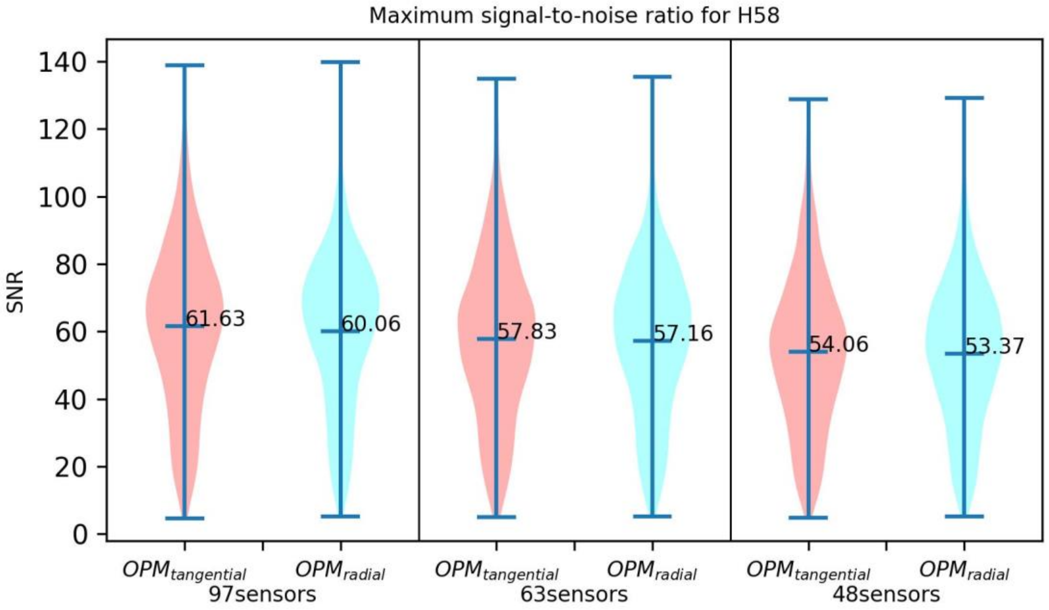

Maximum signal-to-noise ratio for H58
SNR
140
120
100
80
60
40
20
0
61.63
60.06
57.83
57.16
54.06
53.37
OPMtangential
OPMradial
97sensors
63sensors
48sensors

A

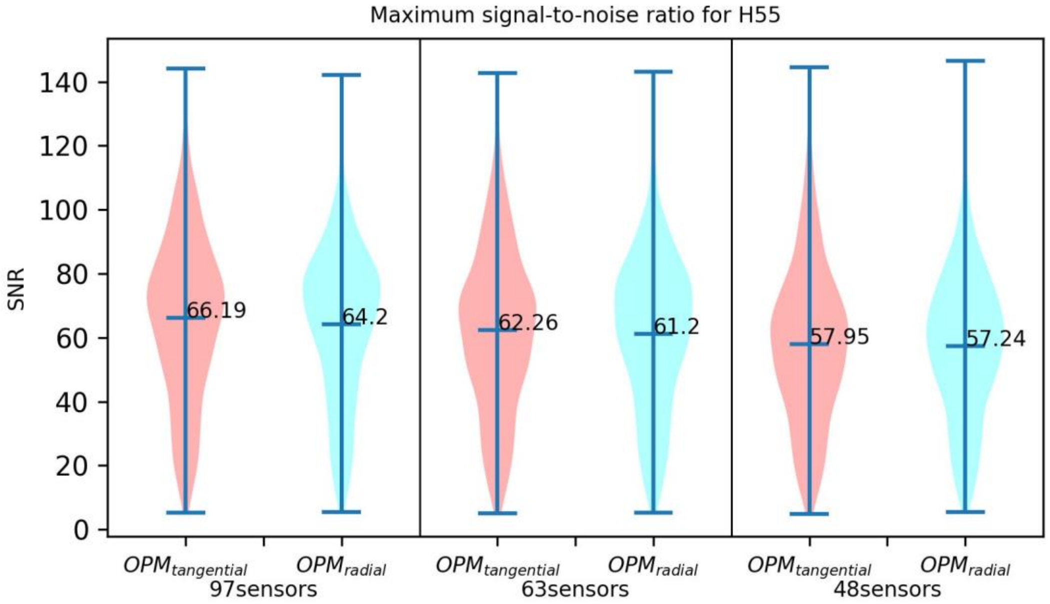

Maximum signal-to-noise ratio for H55
SNR
140
120
100
80
60
40
20
0
66.19
64.2
62.26
61.2
57.95
57.24
OPMtangential
OPMradial
97sensors
63sensors
48sensors

B

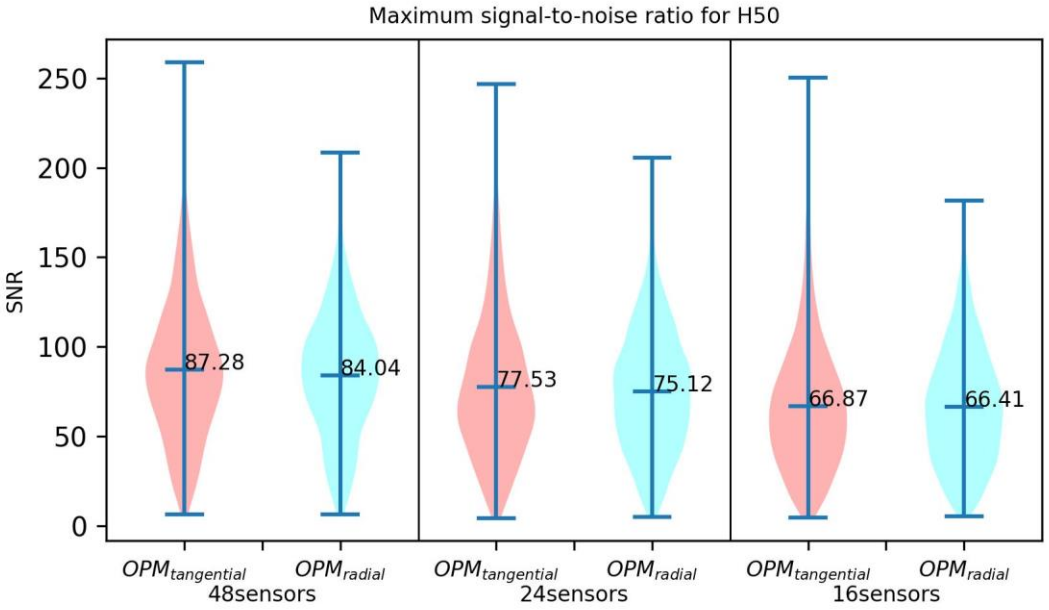

C

Supplementary Figure 1. Maximum SNR distribution computed on radial axes only or tangential axes only, for the H58 (A), H55 (B) and H50 (C) head sizes, for the different sensors arrays from left to right, from 97 to 48 sensors (H58 and H55) or from 48 to 16 sensors (H50). Label "OPM" is referring to ^{4}He OPM MEG. For each head size, axis used in the simulation are shown: the tangential ($OPM_{tangential}$, red) or radial (OPM_{radial}, blue) axis individually. The mean of each distribution is shown at the horizontal marker.

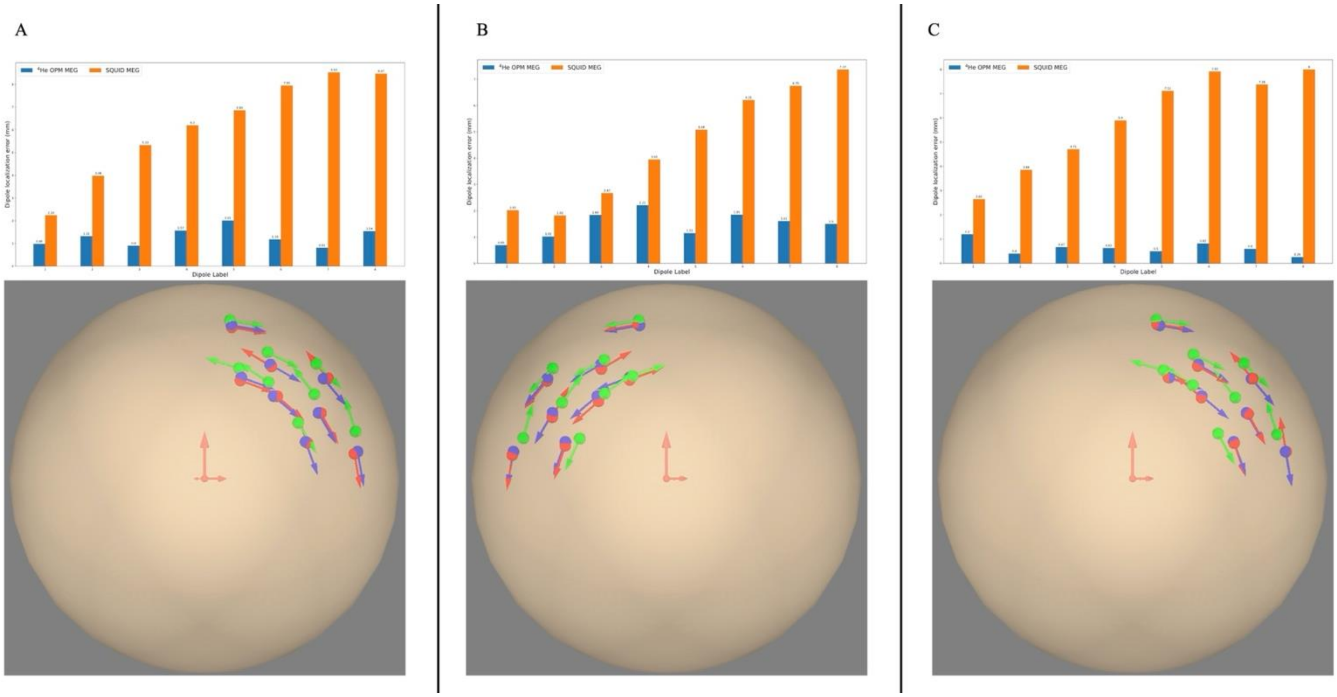

Supplementary Figure 2. Phantom dipole localization error with: Top: DLE (mm) obtained with ^{4}He OPM MEG (blue) and SQUID MEG (orange) for each dipole for the PCB 1 (A), PCB 2 (B) and PCB 4 (C). Bottom: localizations of actual dipoles (blue arrows) and estimated

dipoles for the ^{4}He OPM MEG (red arrows) and for the SQUID MEG (green arrow) for the PCB 1 (A), PCB 2 (B) and PCB 4 (C).
